# Phenotypic Responses to and Genetic Architecture of Sterility Following Exposure to Sub-Lethal Temperature During Development

**DOI:** 10.3389/fgene.2020.00573

**Published:** 2020-06-03

**Authors:** Martyna K. Zwoinska, Leonor R. Rodrigues, Jon Slate, Rhonda R. Snook

**Affiliations:** ^1^Department of Zoology, Stockholm University, Stockholm, Sweden; ^2^Department of Animal and Plant Sciences, University of Sheffield, Sheffield, United Kingdom

**Keywords:** climate change, heat stress, thermal fertility limits, heat-induced male sterility, *Drosophila* Genetic Reference Panel, *Drosophila melanogaster*, GWAS, phenotypic plasticity

## Abstract

Thermal tolerance range, based on temperatures that result in incapacitating effects, influences species’ distributions and has been used to predict species’ response to increasing temperature. Reproductive performance may also be negatively affected at less extreme temperatures, but such sublethal heat-induced sterility has been relatively ignored in studies addressing the potential effects of, and ability of species’ to respond to, predicted climate warming. The few studies examining the link between increased temperature and reproductive performance typically focus on adults, although effects can vary between life history stages. Here we assessed how sublethal heat stress during development impacted subsequent adult fertility and its plasticity, both of which can provide the raw material for evolutionary responses to increased temperature. We quantified phenotypic and genetic variation in fertility of *Drosophila melanogaster* reared at standardized densities in three temperatures (25, 27, and 29°C) from a set of lines of the *Drosophila* Genetic Reference Panel (DGRP). We found little phenotypic variation at the two lower temperatures with more variation at the highest temperature and for plasticity. Males were more affected than females. Despite reasonably large broad-sense heritabilities, a genome-wide association study found little evidence for additive genetic variance and no genetic variants were robustly linked with reproductive performance at specific temperatures or for phenotypic plasticity. We compared results on heat-induced male sterility with other DGRP results on relevant fitness traits measured after abiotic stress and found an association between male susceptibility to sterility and male lifespan reduction following oxidative stress. Our results suggest that sublethal stress during development has profound negative consequences on male adult reproduction, but despite phenotypic variation in a population for this response, there is limited evolutionary potential, either through adaptation to a specific developmental temperature or plasticity in response to developmental heat-induced sterility.

## Introduction

An increase in mean temperatures and temperature variation associated with ongoing climate change threatens biodiversity (Pachauri et al., 2015). Ectotherms play a critical role in ecosystem functioning (Weisser and Siemann, 2008) and can be particularly vulnerable to the effects of climate change because their physiology and biochemistry depend directly upon ambient temperatures (Hochachka and Somero, 2002; Deutsch et al., 2008). Climate change risk assessments are frequently based on quantification of thermal parameters (Deutsch et al., 2008; Sinclair et al., 2016; Kellermann and van Heerwaarden, 2019), such as thermal tolerance (e.g., either critical tolerance or lethal temperatures, such as lower temperatures (CTmin) and higher temperatures (CTmax), representing a species lower and upper operational temperature), and thermal performance curves, e.g., reaction norms in which individuals are exposed to different temperatures until performance fails at CTmin and CTmax. These parameters are associated with latitudinal species’ range distributions (Addo-Bediako et al., 2000; Kellermann et al., 2012; Overgaard et al., 2014). For ectotherms, thermal performance is skewed such that performance drops sharply at increasing, but not decreasing, temperatures. Upper critical thermal limits of terrestrial ectotherms show considerably less geographical variation than lower limits (Addo-Bediako et al., 2000; Deutsch et al., 2008; Kellermann et al., 2012) and many species are thought to operate close to their upper performance limits (Huey et al., 2012; van Heerwaarden et al., 2016). Although some *Drosophila* species have latitudinal clines of CTmax (Castañeda et al., 2015; O’Brien et al., 2017), suggesting the ability to locally adapt to varying temperatures, most evidence on the evolutionary potential for increasing heat tolerance (e.g., shifting critical thermal maximum) suggests limited genetic variability to respond to selection (Castañeda et al., 2019; Kellermann and van Heerwaarden, 2019). Phenotypic plasticity of thermal tolerance parameters, such as CTmax, may be critical to species persistence but many species appear to have a small capacity to shift CTmax via phenotypic plasticity (Sørensen et al., 2016; Kellermann and Sgrò, 2018). Because the capacity for adaptation to climate warming will depend on the underlying genetic architecture and the extent to which adaptation and plasticity contributes to responses to climate warming, these patterns indicate much concern about the consequences of a warming climate on ectotherm species’ distributions and persistence.

Thermal performance measures used in these analyses commonly are based on performance proxies of survival, such as when respiration or movement stop, or death occurs (Kellermann and van Heerwaarden, 2019; Walsh et al., 2019b). However, reproductive performance, such as fertility, can be negatively affected by temperatures that are neither incapacitating nor lethal (Jørgensen et al., 2006; Austin et al., 2013; Kjærsgaard et al., 2013; Manenti et al., 2014; Kingsolver et al., 2015; Porcelli et al., 2017; Sales et al., 2018; Saxon et al., 2018). This raises concerns over whether predictions for species’ responses to increased temperature based on critical thermal limits alone may be too conservative (Walsh et al., 2019b). Complementary to studies of critical thermal limits, knowledge of thermal fertility limits, the reproductive equivalent of critical thermal limits, is necessary to assess the extent of the problem. Yet, few studies have systematically determined either the upper temperatures at which reproduction fails or described the thermal fertility reaction norm within a population. Likewise, to our knowledge, there have been no studies determining the underlying genetic architecture of this response.

While most studies predicting species’ response to climate change incorporate only data from the adult stage, thermal sensitivity may vary across different life cycle stages (Kingsolver et al., 2011; Sinclair et al., 2016; Moghadam et al., 2019). Sublethal but stressful temperatures experienced during juvenile development of *Drosophila subobscura* resulted in fertility loss whereas keeping adults at the same temperature had no negative fertility effect. Likewise, in *Drosophila melanogaster*, the effect of brief high temperature exposure on survival varied across life history stages with adaptive hardening (i.e., previously briefly exposed to high temperatures) more pronounced at juvenile stages (Moghadam et al., 2019). This result suggests that *D. melanogaster* juveniles exhibit higher plasticity in response to temperatures than adults, who can rely to a larger degree on behavioral responses.

Male reproductive performance is thought to be affected by temperature to a greater degree than female reproductive performance because spermatogenesis, which in many insects starts during the juvenile period (Nijhout, 1998), is more thermally sensitive than oogenesis (Setchell, 1998; David et al., 2005; Hansen, 2009). Heat stress experienced during development can render males either temporarily or permanently sterile (Chakir et al., 2002; Araripe et al., 2004; Rohmer, 2004; Vollmer et al., 2004; David et al., 2005; Jørgensen et al., 2006; David, 2008; Pedersen et al., 2011; Porcelli et al., 2017). Even when changes to the male reproductive system are reversible, heat stress can have serious negative consequences for short-lived organisms such as many insects (Sinclair and Roberts, 2005). However, few studies have directly addressed sex-specific thermal sensitivity of reproductive performance, particularly following developmental heat stress (Walsh et al., 2019a).

Here we aim to characterize phenotypic and genetic variation in developmentally heat induced sterility and its plasticity. We used genome-sequenced lines from the *Drosophila* Genetic Reference Panel (DGRP), a set of inbred *D. melanogaster* lines (Mackay et al., 2012; Huang et al., 2014), exposing juveniles to three different temperatures and measuring subsequent fertility in the adult stage. We quantified phenotypic variation and examined the correlation with traits measured on different abiotic stressors that have been published using the panel. We determined the genetic architecture of the reproductive traits and performed a genome-wide association study (GWAS) to identify trait-associated genetic variants. We concentrated on males but, for a smaller subset of lines, we also provide phenotypic data on female reproductive performance.

## Materials and Methods

### Fly Stocks and Maintenance

We used isogenic, genome-sequenced lines from the DGRP, initiated from a natural population from Raleigh, North Carolina that underwent 20 generations of full-sib mating (Mackay et al., 2012; Huang et al., 2014). Climate in Raleigh is humid subtropical, characterized by hot and humid summers with average high temperatures reaching ∼32°C Weather-us.com (2020)^1^. We quantified male fertility responses from 127 DGRP lines. Following determination of high and low performing male lines, we then quantified female fertility responses from 40 lines. We standardized the female (or males for female fertility responses) used as mates across our experiments, using a wild-type Canton Special (CS) strain (gift from Dick Nässel, originally obtained from the Bloomington Stock Center). CS and DGRP stock flies were maintained in standard culture vials using cornmeal medium (10 L: 9 L water, 720 *g* cornmeal, 162 *g* dried yeast, 90 *g* soya flour, 720 *g* malt extract, 360 *g* molasses, 72 *g* agar, 36 mL propionic acid, and 225 mL of 10% Nipagin) at 12−h light:12−h dark cycle at 25°C. Mates for experimental individuals were similarly reared at 25°C throughout development, under controlled density conditions (100 eggs/vial), subsequently collected as virgins under CO_2_ anesthesia, transferred to vials in groups of about 20, stored at 25°C, and were 3–6 day old when used for experiments.

### Responses of Fertility to Developmental Thermal Stress

#### Males

For focal experimental males of each DGRP line, we standardized egg number by placing 2-week old adult flies onto egg laying media (6 *g* agar, 57.5 *g* bread syrup, and 360 mL of water, seeded with 100 μL yeast paste upon drying) for 2–3 days prior to egg collection at 25°C. In the morning on the day of egg collections, flies were transferred onto fresh egg laying media for 2–4 h, eggs collected onto mesh screen (Snook et al., 1994), and groups of ca. 50 eggs were counted and transferred into vials filled with cornmeal medium. Replicate vials were made for each DGRP, with subsets of vials placed into each control temperature incubator (Panasonic MIR-154) set to 25, 27, or 29°C, 12−h light: 12−h dark cycle. Virgin males from each line were collected under CO_2_ anesthesia and transferred into individual vials then stored at 25°C. The day following eclosion, a single virgin control female was added to each vial. Experimental pairs were allowed to interact for 3 days, then removed from vials. Reproductive performance was scored 2 days later as a binomial trait; fertile males produced at least one larva and sterile males did not. We had to run the experiment in blocks. This was due to the large number of DGRP lines assayed (*n* = 127) and because for each line we simultaneously tested fertility in response to three different temperatures, while strictly controlling egg density in vials generating experimental individuals. We ran 13 blocks, each consisting of 7–11 DGRP lines, with most blocks having 10 lines. To account for block variation, we assessed fertility of CS males at 25°C and 29°C in each block. The mean number of individual males/line/temperature was 24.8 (median 28). See Supplementary Table S1 for details of the DGRP lines used, number of flies/line/temperature, and trait values.

#### Females

To determine the consequences of developmental heat stress on female fertility we used a subset (*n* = 40) of tested male lines. This subset represented the lines performing well across all temperatures (“high lines” – 19 lines) and lines performing poorly as temperature increases (“low lines” – 21 lines). Performance category was based on a variety of considerations, but firstly on males’ phenotypic response at 29°C and the slope of response (based on random intercepts and slope model described in the below section “Genome-wide association response variables: temperature-specific reproductive performance and plasticity”), then on other considerations such as the number of replicates contributing to the values, and whether we could obtain sufficient number of individuals before the experiment. We ran the female experiment three times, each consisting of 12–15 DGRP lines averaging 27.39 (median 28) females/line/temperature and CS. See Supplementary Table S1 for details of the DGRP lines used, number of flies/line/temperature, and trait values.

### Statistical Analyses

#### Average Phenotypic Responses

We categorized the effects of each temperature on reproductive performance as binomial; for each mating pair, the reproductive response was either a success (at least one larva was produced) or a failure (no larva produced), taking into account the number of males tested in each line (function *cbind*, in R; see Supplementary Table S1 for data). For each line then we get a proportion of males assayed that are fertile. To address the use of this conservative estimate for temperature-induced impacts on fertility, we fitted binomial mixed-effect models with a logit link function using the *lme4* package in R (Bates et al., 2015). The model for male dataset included temperature as a fixed factor and block and DGRP line as random factors [*cbind (Reproduced, Did not reproduce)* ∼ *Temperature* + *(1| Block)* + *(1| DGRP line)*]. The model for female data set had an additional fixed factor, *Line status*, which indicated whether the line was classified as high or low performing based on the reproductive performance of males [*cbind (Reproduced, Did not reproduce)* ∼ *Temperature × Line status* + *(1| Block)* + *(1| DGRP line)*]. In these models we included CS to account for variation in each block but model fits with CS included did not perform better than model fits without CS included. This is likely because there was little variation across temperatures in CS performance. Thus, to assess the extent to which DGRP responses were repeatable (and therefore potentially impacted by variation between blocks), we analyzed male data from two subsequent experiments we have run only on the high and low lines, with the experimental design exactly the same as here. That is, we have measured male fertility, under the same conditions, in 40 lines, three different times. We used the *corrplot R* package to obtain a matrix of Pearson correlations and the *Hmisc* package to calculate the *p*-values of the correlation between fertility measures for DGRP lines across the three different sampling periods. We found consistent results at 27°C and especially at 29°C, with correlations across these experiments at the higher temperature ranging from 0.73 to 0.88; low repeatability at 25°C is likely a consequence of little variation across lines (Supplementary Table S2). This analysis confirms responses are repeatable, particularly at the highest temperature which is also the most phenotypically variable across the lines. Experimental block was included as a random factor to help account for the non-independence of observations within a single experimental unit (Harrison et al., 2018). *Wolbachia* and inversion status of the DGRP lines used were fitted as fixed factors but inclusion of all of them caused convergence issues. Fitting them individually returned no significant effect.

Models for each temperature treatment were run separately using block and DGRP line as random factors [*cbind (Reproduced, Did not reproduce)* ∼ *(1| Block)* + *(1| DGRP line)*]. The variance components of line and block, along with the residual variance, assumed to be π^2/3^ (Nakagawa and Schielzeth, 2010), of each of these binomial models was used to calculate broad-sense heritabilities, and such that *H*^2^ = *V*_line_/(*V*_line_ + *V*_block_ + *V*_resid_). We note that although the variance explained by line is assumed to be some form of genetic variance (i.e., additive genetic variance, dominance genetic variance, and epistasis or gene-by-environmental interaction), it is not possible to partition the line variance between these different genetic components. We also note that part of the variance explained by block is likely to include some form of genetic variance but it is not possible to partition the part of block variance that would contribute to genetic components of line variance. Thus, the *H*^2^ estimate is likely conservative. We used the package *lsmeans* (Lenth, 2016) to obtain least squares means for each temperature treatment and converted them from the logit scale to obtain predicted probabilities of reproducing at a given temperature.

### Genome-Wide Association Response Variables: Temperature-Specific Reproductive Performance and Plasticity

We initially intended to perform a mixed-model GWAS with binomial response variables using the package GMMAT (Chen et al., 2016; Chen et al., 2019) but sensible results at 25°C and 27°C were not produced, likely because all lines performed almost equally well at these temperatures. Thus, we used line-specific intercepts, as a measurement of temperature-specific reproductive performance, at each temperature and line-specific slopes, as a measure of phenotypic plasticity of reproductive performance. In the random slopes model used to extract line-specific intercepts and slopes, temperature was added as fixed factor, while block and DGRP line were treated as random factors [*cbind (Reproduced, Did not reproduce)* ∼ *Temperature* + *(1| Block)* + *(Temperature| DGRP line)*]. Temperature was a continuous variable in this model and was centered at each temperature treatment (25, 27, or 29°C) to extract treatment-specific intercepts. The random factor of the DGRP line *(Temperature| DGRP line)* allowed for the effect of temperature to vary between the lines and provided the line-specific slope estimate. We extracted the model intercepts and slopes with the *coef* command from the binomial random slope model with a logit link function fitted using the *lme4* package (Bates et al., 2015). The model terms for each line’s slope and intercept were continuous, making them more tractable for GWAS than if they were binomial variables (see Supplementary Table S1 for estimates, Supplementary Figure S1 for temperature-specific and slope values, and Supplementary Figure S2 for frequency distribution of values).

### SNP Filtering and Quality Control

Quality control of the genomic data was performed in Plink v1.9 (Purcell et al., 2007; Chang et al., 2015). We set the minor allele frequency threshold (MAF) to be at least 5% and we filtered out all variants that were missing in more than 10% of lines (–*geno* in Plink). The rate of genotype missingness (–*mind* in Plink) for each line was set to be not more than 15%, which ensured the retention of all 127 phenotyped lines. A total of 1,465,358 variants were retained after quality control.

### SNP-Based Heritability

We estimated the proportion of variance for male phenotypes explained by all genetic variants, the SNP-based heritability (sensu Yang et al., 2010), using the GREML approach implemented in the GCTA software (Yang et al., 2011). GREML uses a genetic relatedness matrix (GRM) to perform a marker-based animal model to measure the proportion of variation explained by additive genetic effects. Here the GRM was created using autosomal markers only (1,230,417 variants). Male traits were phenotypic plasticity of fertility in response to developmental heat stress (slope) and fertility at each temperature (intercept for each temperature).

### Genome-Wide Association Analyses

For association tests, we used GMMAT, implemented in R (Chen et al., 2016, 2019). First, we fitted linear mixed models to adjusted phenotype data obtained from the mixed models described above. GWAS phenotypes were the model intercepts (male fertility at each temperature) and slopes (the plasticity of male fertility) with family set to *gaussian* and the link function set to *identity*. *Wolbachia* status of each DGRP line and 5 major inversions present in the DGRP panel [*In(2L)t*, *In(2R)NS*, *In(3R)P*, *In(3R)K*, and *In(3R)Mo*] were included as fixed factors. To account for cryptic genetic relatedness, we fitted a centered GRM, created using the GEMMA package, as a random factor in our model. Only the autosomal markers were used to create this matrix. GMMAT performs the GWAS by adding each SNP, in turn, to the model to test for associations between genotype and phenotype. Because some of the genetic markers will be in linkage disequilibrium with one another, we estimated the effective number of tests (*M*_*e*_) in the GWAS, using the Genetic Type 1 error calculator (Li et al., 2012). *M*_*e*_ was 722,833 which means that a genome-wide significance threshold at *P* < 0.05 requires a SNP to be nominally significant at *P* = 6.92 × 10^–8^.

### Correlations With Other DGRP Datasets

We used the *corrplot R* package to obtain and plot a matrix of Pearson correlations and the *Hmisc* package to calculate the *p*-values of each correlation between fertility measures for males and other stress-related traits measured in the DGRP. Because phenotypic variation in fertility was highest among the lines at 29°C, and because the random slope and intercept model corrects for block effects, we compared the intercept of fertility at 29°C against traits associated with other abiotic stressors: chill coma resistance and starvation resistance (Mackay et al., 2012), desiccation tolerance (Rajpurohit et al., 2018), CTmax (Rolandi et al., 2018), and two measures of oxidative stress based on two different oxidative stress-inducing agents, paraquat and menadione sodium bisulfite (MSB) resistance (Weber et al., 2012). Traits were analyzed based on male trait value/line for all comparisons.

## Results

### Phenotypic Response

#### Males

**FIGURE 1:**
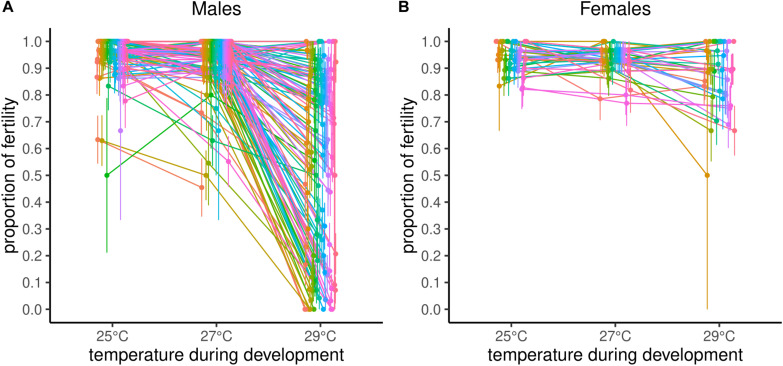
Natural genetic variation in fertility of males **(A)** and females **(B)** at three different developmental temperatures.

Reproductive performance was significantly negatively affected as developmental temperature increased, with 25°C as the least affected, 27°C intermediate, and 29°C the most affected (Figure 1A and Table 1). Impact of different temperatures resulted in probabilities of reproducing, derived from least square means, of 0.98 at 25°C, 0.97 at 27°C, and 0.55 at 29°C. Block effect explained about 0.26 of variance of the model (σ^2^_Block_ divided by σ^2^_Block_ + σ^2^_Line_ + σ^2^_Residual_) and DGRP line explained about 0.20 of variance (σ^2^_Line_ divided by σ^2^_Block_ + σ^2^_Line_ + σ^2^_Residual_, Table 1). Recall, however, that among high and low performing lines, repeatability of results was ca. 80% at 29°C, suggesting block variance is at least partially a consequence of biological variation in the lines tested in any given block. Broad-sense heritability, determined by variance explained by the DGRP line, differed between temperature treatments but was highest at 29°C where there was the most phenotypic variability among the lines (Table 2).

**TABLE 1:** Generalized mixed-effect model on male reproductive performance (binomial; number of successfully reproducing and unsuccessful females for high and low performing male lines) following developmental heat stress at 25, 27, and 29°C.

	Estimate	Std. Error	*Z* value	*P* value
**Fixed effects**				
Intercept (Temperature 25°C)	4.16	0.33	12.48	<0.001
Temperature (27°C)	–0.63	0.13	–5.035	<0.001
Temperature (29°C)	–3.96	0.12	–33.058	<0.001

	**Parameter**	**Variance**	**Std. Dev.**	

**Random effects**				
Block	Intercept	1.61	1.077	
Line	Intercept	1.21	1.10	

	**Estimate**	**Std. Error**	***Z* value**	***P* value**

***Post hoc* contrasts**				
29°C–27°C	–3.33	0.10	–32.39	<0.001
Residual variance of the model: 3.29				
Residual deviance: 594.842 on 367 degrees of freedom (ratio: 1.621)				

*Post hoc contrast between 27°C and 29°C provided as this is not directly tested in the main model.*

**TABLE 2:** Heritability measurements for male fertility following developmental heat stress at 25, 27, and 29°C.

*H*^2^ Estimate	25°C	27°C	29°C	Slope
σ^2^ for DGRP line	0.74	0.84	2.20	
σ^2^ total	4.03	4.13	5.49	
*H*^2^	0.18	0.20	0.40	

**SNP-based heritability**	**25°C variance ± SE**	**27°C variance ± SE**	**29°C variance ± SE**	**Slope variance ± SE**

**Source**				
*V*_g_	0.00 ± 0.58	0.00 ± 0.12	0.00 ± 0.67	0.00 ± 0.11
*V*_e_	2.33 ± 1.20	0.41 ± 0.24	1.83 ± 1.35	0.40 ± 0.23
*V*_p_	2.33 ± 0.65	0.41 ± 0.13	1.83 ± 0.70	0.40 ± 0.12
*V*_g_/*V*_p_	0.00 ± 0.25	0.00 ± 0.28	0.00 ± 0.37	0.00 ± 0.28

*Broad-sense heritabilities (H^2^) calculated from between-line variances for each temperature derived from the mixed effect model (see Table 1) and SNP-based heritability estimates on the intercepts of each temperature and slope obtained using the GCTA software and the GREML approach.*

Intercept values, used for GWAS, resulted in similar probabilities of reproducing, based on binomial data; 1 (0.995) at 25°C, 0.94 at 27°C, and 0.59 at 29°C (values on a logit scale based on intercept means of 5.36, 2.87, and 0.38 at 25, 27, and 29°C, respectively; Supplementary Table S3). The slope of reproductive performance declines by the proportion of 0.22 across the treatments (value on a logit scale was –1.25; Supplementary Table S3). Note that the model for 25°C returned warnings about convergence failure, but generated estimate outputs.

#### Females

Temperature also significantly affected female reproductive performance, although unlike males, females reproductive performance did not differ between 25°C and 27°C (Figure 1B and Table 3). Reproductive performance of males from the same line (Line status) was not a significant predictor in the female model (*z* = −1.40, *P* = 0.16) indicating little or no association between male and female fertility in response to thermal stress. Probabilities of reproducing at different temperatures, derived from least square means, were 0.95 for 25°C, 0.93 for 27°C, 0.90 for 29°C, substantially higher at 29°C than for males. Broad-sense heritabilities returned singular fits except for 27°C treatment, estimated to be 0.01.

**TABLE 3:** Generalized mixed-effect model on female reproductive performance (binomial; number of successfully reproducing and unsuccessful females for high and low performing male lines) following developmental heat stress at 25, 27, and 29°C.

	Estimate	Std. Error	*Z* value	*P* value
**Fixed effects**				
Intercept (Temperature 25°C, High)	3.02	0.23	13.28	<0.001
Temperature (27°C)	–0.19	0.17	–1.12	0.26
Temperature (29°C)	–0.64	0.16	–4.01	<0.001
Line status (Low)	–0.32	0.23	–1.40	0.16

	**Parameter**	**Variance**	**Std. Dev.**	

**Random effects**				
Block	Intercept	0.32	0.56	
Line	Intercept	0.04	0.19	

	**Estimate**	**Std. Error**	***Z* value**	***P* value**

***Post hoc* contrasts**				
29°C–27°C	–0.45	0.15	–2.93	0.003
Residual variance of the model: 3.29				
Residual deviance: 117.983 on 114 degrees of freedom (ratio: 1.035)				

*Post hoc contrast between 27°C and 29°C provided as this is not directly tested in the main model.*

### SNP-Based Heritability

SNP-based heritability analyses in GCTA revealed narrow-sense heritability and additive genetic variance of 0 for all traits analyzed (Table 2), although it should be noted that the standard errors around these estimates were quite large, meaning that the possibility of some genetic variance in these traits cannot be ruled out.

### Genome-Wide Association Analyses

**FIGURE 2:**
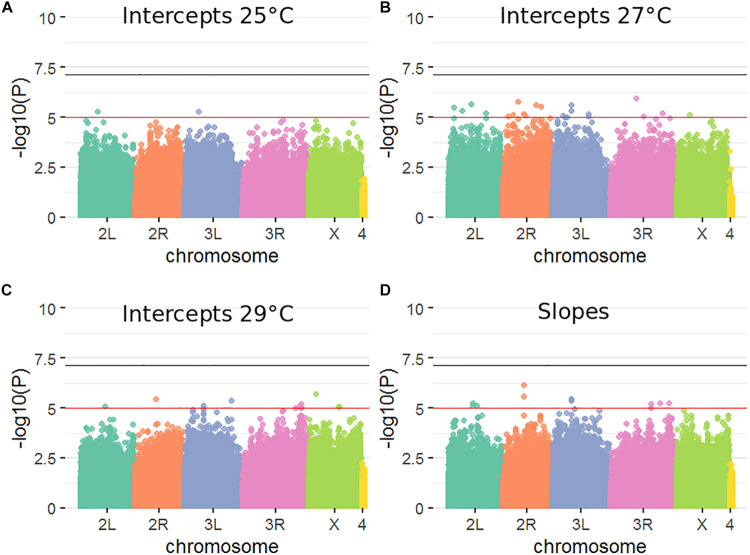
Genetic variants influencing male fertility at three different temperatures and the slope of response. **(A–D)**. Manhattan plots corresponding to four GWAS analyses performed. Horizontal lines are *p*-value = 1 × 10^−5^ (red) and *p*-value = 6.92 × 10^−8^ (black), where 1 × 10^−5^ corresponds to -log(10) of 5 and 6.92 × 10^−8^ corresponds to -log(10) of 7.16.

Standard practice for GWAS analyses in the DGRP panel (Mackay et al., 2012) is to use a nominal *p* value of *P* < 1 × 10^–5^ threshold for reporting significant SNPs (indicated as a red line in Figure 2). The number of variants meeting this threshold was: two at 25°C, 21 at 27°C, 10 at 29°C, and 13 for plasticity (Figure 2). Three variants overlapped between the 29°C and slope analysis (see Supplementary Table S5 for list of nominally significant variants). Importantly, with 1,465,358 variants analyzed and a *p* value threshold of 1 × 10^–5^ one would expect ∼15 significantly associated variants by chance alone. Quantile-quantile plots (Supplementary Figure S3) further illustrated no enrichment of associations exceeding the *P* < 1 × 10^–5^ threshold. No variant passed a more stringent significance threshold, for instance one based on an *M*_*e*_ of 722,833 which is equivalent to a *p* value of 6.92 × 10^–8^ or -log10(*p*) ∼ 7.16 (indicated as a black line in Figure 2). The lowest *p* values were in the range of 1.16 × 10^–6^, corresponding to -log10(*p*) ∼ 5.94. Thus there is no statistical support for the claim that any of the variants that passed the *p* < 10^–5^ threshold represents a true positive finding. It is therefore unsurprising that GO enrichment analysis (Ashburner et al., 2000; The Gene Ontology Consortium, 2019) revealed no significant enrichment for any of the measured phenotypes (not shown).

### Comparison With Other DGRP Datasets

There was a significant positive correlation between MSB resistance (survival time) and fertility at 29°C but all other comparisons between male fertility during developmental heat stress across lines and other traits responsive to abiotic stressors in the DGRP were not significant (Table 4).

**TABLE 4:** Phenotypic co-variation between male fertility at 29°C (intercept extracted from the random slopes model, corrected for block effect) and other fitness traits following abiotic stress using Pearson-correlation coefficient.

Environmentally relevant trait	Correlation coefficient	*P*-value	Nr lines
Chill coma recovery	0.132	0.199	96
Starvation resistance	0.084	0.403	102
Desiccation tolerance	0.080	0.413	108
CTmax	–0.187	0.417	21
Paraquat resistance	0.184	0.064	102
MSB resistance	0.210	0.034	102

*The number of lines (nr lines) that overlap between this fertility study and the other variables ranged from 21 to 108. All comparisons were made using data from only males. NB: similar results were found using the fertility slope. Data was obtained from each reference’s Supplementary Material: Mackay et al., 2012, Table S20 (Chill Coma Recovery, Starvation Resistance); Rajpurohit et al., 2018, Supplementary Table S3 (Desiccation Tolerance); Rolandi et al., 2018, Supplementary Table S1 (CTmax); and Weber et al., 2012, Supplementary Table S1 (Paraquat and MSB Resistance).*

## Discussion

Understanding the consequences of increasing temperature on sex-specific fertility effects, and the evolutionary and plastic responses of natural populations to thermal challenges, will help improve predictions for species’ persistence. In this study, we determined the impact on adult fertility of sublethal heat stress following developmental exposure to three different temperatures, determined the thermal reaction norm, and assessed genetic architecture of measured traits in a mapped population of *D. melanogaster*. We found males were affected to a larger degree than females by higher developmental temperatures, and the difference was particularly striking at the highest temperature. The average male fertility for each DGRP line, for both 29°C and for the slope of fertility across all temperatures, was correlated with previous DGRP results on male survival after oxidative stress (Weber et al., 2012). Despite significant phenotypic variance in male thermal fertility limits at 29°C and in the slope of response across all temperatures, we found little evidence of heritable genetic variation for these reproductive traits. The number of genetic variants significantly associated with the traits analyzed at the nominal *p* value threshold of 10^–5^ did not exceed what would be expected by chance alone. We discuss our results in light of what may be driving the sterility effect, the genetic architecture of fitness-related traits in light of previous DGRP results, and the impact that temperature-induced sterility may have on population persistence.

Sex-specific thermal sensitivity was observed. Female fertility was not assessed in all DGRP lines that male reproductive performance was measured in as early results suggested females were not as affected. However, we found there was no effect of whether male reproductive performance was either relatively insensitive (high performing), or sensitive (low performing) to developmental temperature on female fertility, indicating male and female reproductive performance in response to developmental heat-stress is unlinked. While both sexes showed increased between-line variation after developing at 29°C, male reproductive performance was affected to a much larger degree, both with respect to estimates of thermal fertility limit and its phenotypic plasticity (slope of the reaction norm), than female reproductive performance. Similar sex-specific results were recently reported following adult heat stress in the red flour beetle *Tribolium castaneum* (Sales et al., 2018).

We speculate the larger male effect is due to the high thermal sensitivity of spermatogenesis, which in many insects starts during development. We previously found that *D. subobscura* males had reduced sperm motility after experiencing sublethal heat stress during development (Porcelli et al., 2017). Heat wave exposure in adult males in *T. castaneum* caused reduced sperm production and sperm viability (Sales et al., 2018). Thus, sublethal heat stress has effects on sperm quantity and quality (Snook, 2005). Intriguingly, we found that DGRP lines that were less sensitive to heat-induced sterility lived longer following exposure to MSB, an oxidative stress-inducing chemical agent. Oxidative stress is strongly linked with the production of reactive oxygen species (ROS) and is considered to be a main cause of male infertility, causing damage to sperm membranes that impairs sperm-egg interactions, reduces ejaculate quality, including sperm velocity, and can cause sperm DNA damage that also negatively impacts fertilization (Mora et al., 2017). High temperature increases metabolism and therefore increases ROS production (dos Hamilton et al., 2016) and GWAS indicates an association between SNPs in antioxidant genes and male infertility (Yu and Huang, 2015). Future work will assess directly the relationship between developmental heat-induced sterility, consequences to sperm quantity and quality, and the relationship with ROS in *Drosophila*.

CTmax values have been used to assess species consequences to future warming. Previous work on the DGRP has measured adult CTmax which ranged from ca. 40.1°C to 41.5°C (Rolandi et al., 2018). We found no correlation between developmentally heat-induced sterility in this study and adult CTmax (Rolandi et al., 2018). This may be because there were only 21 lines that overlapped between studies and CTmax of those lines did not vary substantially. Regardless, DGRP CTmax temperatures are substantially higher than temperatures that cause lowered male fertility. Our original experimental design included a 31°C temperature treatment to more completely describe fertility limits in this population but, at this higher temperature, substantial juvenile mortality was observed [matching previous descriptions of other *D. melanogaster* populations from temperate collections; (David et al., 2005)]. The comparison between these studies suggest that developmental heat stress, relative to adult heat stress, could have considerable negative impacts on population persistence. This, however, depends on whether future temperatures are expected to go above 29°C for extended periods of time during juvenile development and from which they cannot escape. Rolandi et al. (2018) compared historic climatic records (1980–2005) from Raleigh North Carolina, where the DGRP originated, and found only 10 days above the adult CTmax, but future climate projections (2045–2070) based on the RCP6.0 emissions scenario predicted an increase to 243 days of extreme high temperatures above CTmax. Together, these results suggest that heat-induced sterility during development occurs at temperatures substantially lower than adult CTmax (i.e., those temperatures used to project species response to climate change), and that future temperature regimes are likely to frequently reach temperatures that result in developmentally-induced sterility. Estimates of fertility here were based on binomial quantification, in which producing one larva would count as a male being fertile. This is a conservative estimate of the consequences of developmental heat stress on adult reproduction. Our impression after assaying ca. 10000 males in this study, and based on an experiment we are currently conducting, is that progeny number is substantially reduced at 29°C, even for lines characterized as being fertile. Thus, it is likely that the potential consequences of exposure to sublethal heat stress during development on adult fertility we document here is conservative.

The extent to which the population can respond to temperature selection is critical to determine as this will impact population persistence. Estimates of additive genetic variance and heritability for temperature-specific fertility effects and its phenotypic plasticity do not give cause for optimism. We found little to no additive genetic variance or heritability using SNP-based animal models and no significant SNPs were detected via GWAS. The DGRP can only be used to reliably detect genetic variants of moderate effects, and the mapping power of the panel is considered to be low because of a relatively small number of lines (Turner et al., 2013; Long et al., 2014; Mackay and Huang, 2018). There appears to be epistatic interactions impacting the genetic architecture of quantitative traits in the DGRP (Huang et al., 2012; Shorter et al., 2015); epistatic interactions are not detectable in the analyses we performed as they are only designed to identify additive genetic variation (Yang et al., 2011; Evans et al., 2018). However, the larger estimate of broad-sense heritability compared to narrow-sense heritability suggests that some non-additive genetic variance, possibly due to epistasis, is present. The influence of epistasis on trait expression in these lines has been suggested for several traits (Huang et al., 2012; Shorter et al., 2015; Huang and Mackay, 2016).

Heritability estimates are sensitive to environmental conditions (Hoffmann and Parsons 191). Low heritability estimates for thermal performance traits has been suggested to be a function of the intensity and duration of the thermal treatment (Castañeda et al., 2019). In some studies, increasing the length of the thermal assay lowers heritability, perhaps because additional stress factors (e.g., resource depletion, cellular damage, and dessication resistance), arising under chronic but not acute stress, increase environmental variance (Mitchell and Hoffmann, 2010; Castañeda et al., 2019). However, we find increased broad-sense heritability at higher temperatures with no correlation between heat-induced sterility and other environmental stress factors such as desiccation resistance that may contribute to environmental variance. Previous reviews have described examples of heritability being greatest in stressful conditions (Hoffmann and Parsons, 1991; Hoffmann and Merilä, 1999; Charmantier and Garant, 2005) and in our experiment genetic variation is revealed at the most stressful temperature of 29°C.

Heritability estimates are also impacted by how close the trait is to fitness. We assayed fertility *per se*, a trait intimately related to fitness. Other life history traits closely linked to fitness exhibit lower narrow-sense heritabilities than morphological or physiological traits (Mousseau and Roff, 1987). While low heritabilities can result from high levels of residual genetic variance, rather than low levels of additive genetic variance *per se* (Houle, 1992), numerous studies have found very low levels of additive genetic variance for fitness, and/or fertility (Kruuk et al., 2000; Teplitsky et al., 2009; McFarlane et al., 2014; Sztepanacz and Blows, 2015; Noble et al., 2017). This includes data on *D. melanogaster* outbred and inbred populations (Hughes, 1995a, b; Snoke and Promislow, 2003). Low to zero additive genetic variation, but high dominance genetic variance, for fitness-linked traits has been found in *D. serrata* (Sztepanacz and Blows, 2015), and a study using a *C. elegans* mapping panel of recombinant inbred lines found estimates of the heritability of fertility to not be significantly different from 0. Instead, around 40% of variance in fertility was explained by epistasis (Noble et al., 2017). The study concluded that numerous small-effect epistatic interactions explained non-additive genetic variation in fitness-related traits in this population (Noble et al., 2017), similar to findings on the genetic architecture of quantitative traits in the DGRP (Huang et al., 2012; Mackay and Huang, 2018).

Genome-wide association study analyses did not identify any SNPs that were genome-wide significant for heat-induced sterility and Q-Q plots did not reveal an excess of nominally significant SNPs at lower thresholds. Many DGRP GWAS papers show evidence for a modest excess of loci with *p* values below the 1 × 10^–5^ threshold, suggesting an enrichment of true positive associations (Mackay and Huang, 2018). However, comparisons between genetic variants discovered using the DGRP and other mapping panels or populations rarely reveal overlapping loci (Huang et al., 2012; Swarup et al., 2013; Morozova et al., 2015; Najarro et al., 2015, 2017; Shorter et al., 2015; Carbone et al., 2016; Rajpurohit et al., 2018; Everman et al., 2019). While we report any loci significant at *P* < 1 × 10^–5^ in Supplementary Table S5, we place a caveat that many or perhaps all of these associations are likely to be false positives.

In summary, we showed that male fertility was less thermally tolerant than female fertility, and that males exhibited within-population variation in the response of fertility to sublethal heat stress during development and in phenotypic plasticity of this response. Lines in which males were susceptible to heat-induced sterility were also more susceptible to oxidative stress and oxidative stress has known negative consequences on sperm quantity and quality. Despite a moderate broad-sense heritability at 29°C, we found no evidence of additive genetic variation although some non-additive genetic variation may be present. Likewise, we observed no genetic variants that could be robustly associated with either temperature-specific fertility consequences, even at the most stressful temperature tested, or its plasticity. Future climate scenarios predict increased likelihood for temperatures that could result in at least portions of the population becoming sterile, at temperatures well below those resulting in reduced performance associated with survival, and our current measure of the impact of developmentally-induced sterility is conservative. Therefore, the impact of thermal fertility limits on population persistence under future climate scenarios will need to be considered to help predict responses to increased temperatures.

## Data Availability Statement

All datasets generated and analyzed for this study are included in Supplementary Table S1. These data are also published on Figshare: https://su.figshare.com/articles/Zwoinska_et_al_2020_Heat-induced_sterility_data/12248576/1.

## Author Contributions

RS conceived the experiments. MZ and LR collected the data. The data was analyzed by ZW, JS, and LR. MZ and RS wrote the manuscript with contributions from LR and JS. All authors agreed on the final version of the manuscript.

## Conflict of Interest

The authors declare that the research was conducted in the absence of any commercial or financial relationships that could be construed as a potential conflict of interest.

## Footnotes

1 https://www.weather-us.com/en/north-carolina-usa/raleigh-climate?c,mm,mb,km#temperature
