# Supplementary material for: Phenotypic Responses to and Genetic Architecture of Sterility Following Exposure to Sub-Lethal Temperature During Development
Source: Front Genet. 2020 Jun 3;11:573. doi: 10.3389/fgene.2020.00573 (PMC7283914; doi:10.3389/fgene.2020.00573)
Supplement: Supplementary file 1 [file Data_Sheet_1.zip › Tables S2 - S5 and Figures.docx]

**Supplemental figures and tables**


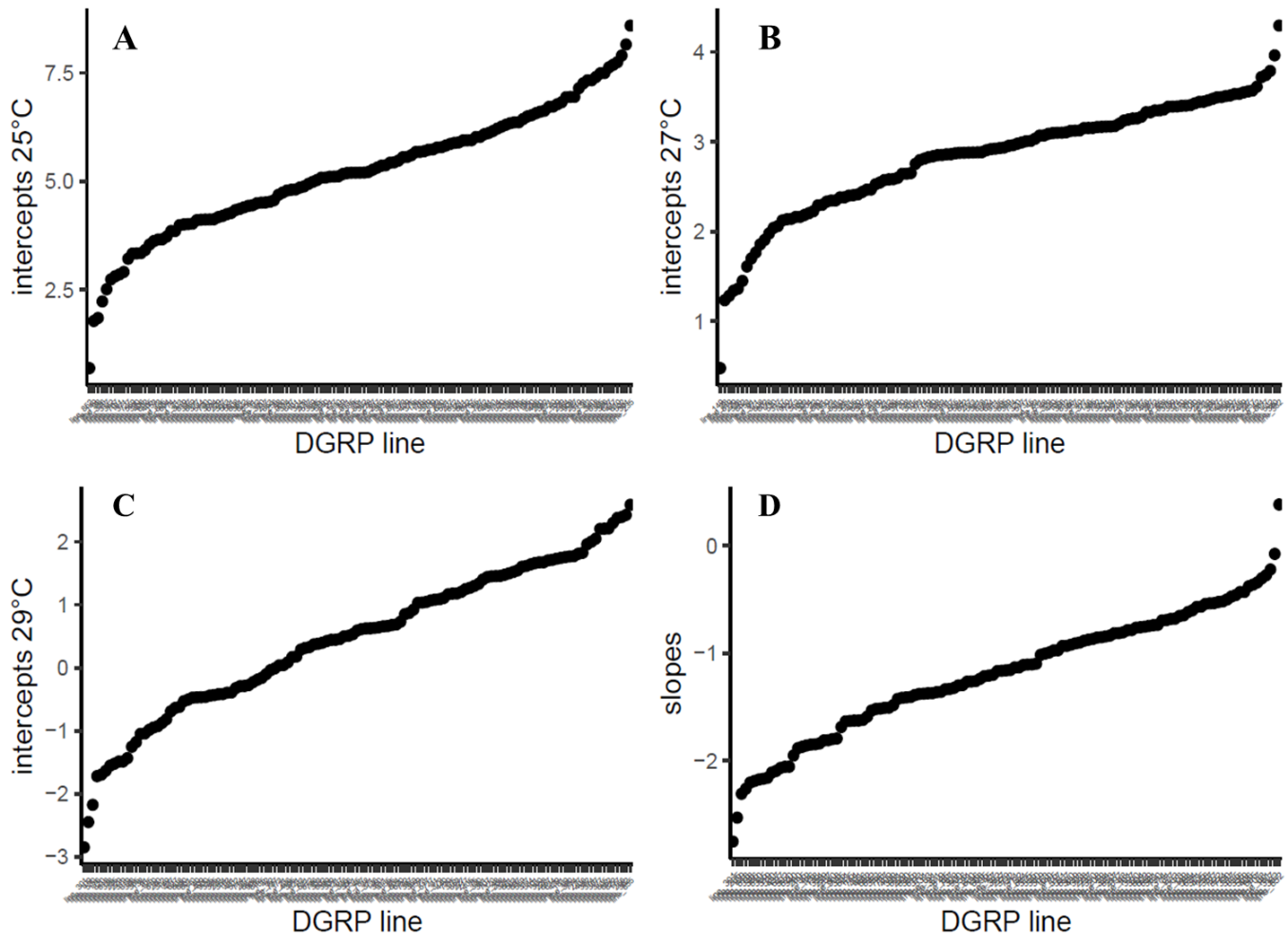


**Supplementary Figure 1** Intercept and slope values of each DGRP line tested for male fertility following developmental heat stress at **A** 25°C **B** 27°C **C** 29°C, and **D** plasticity of response.

**
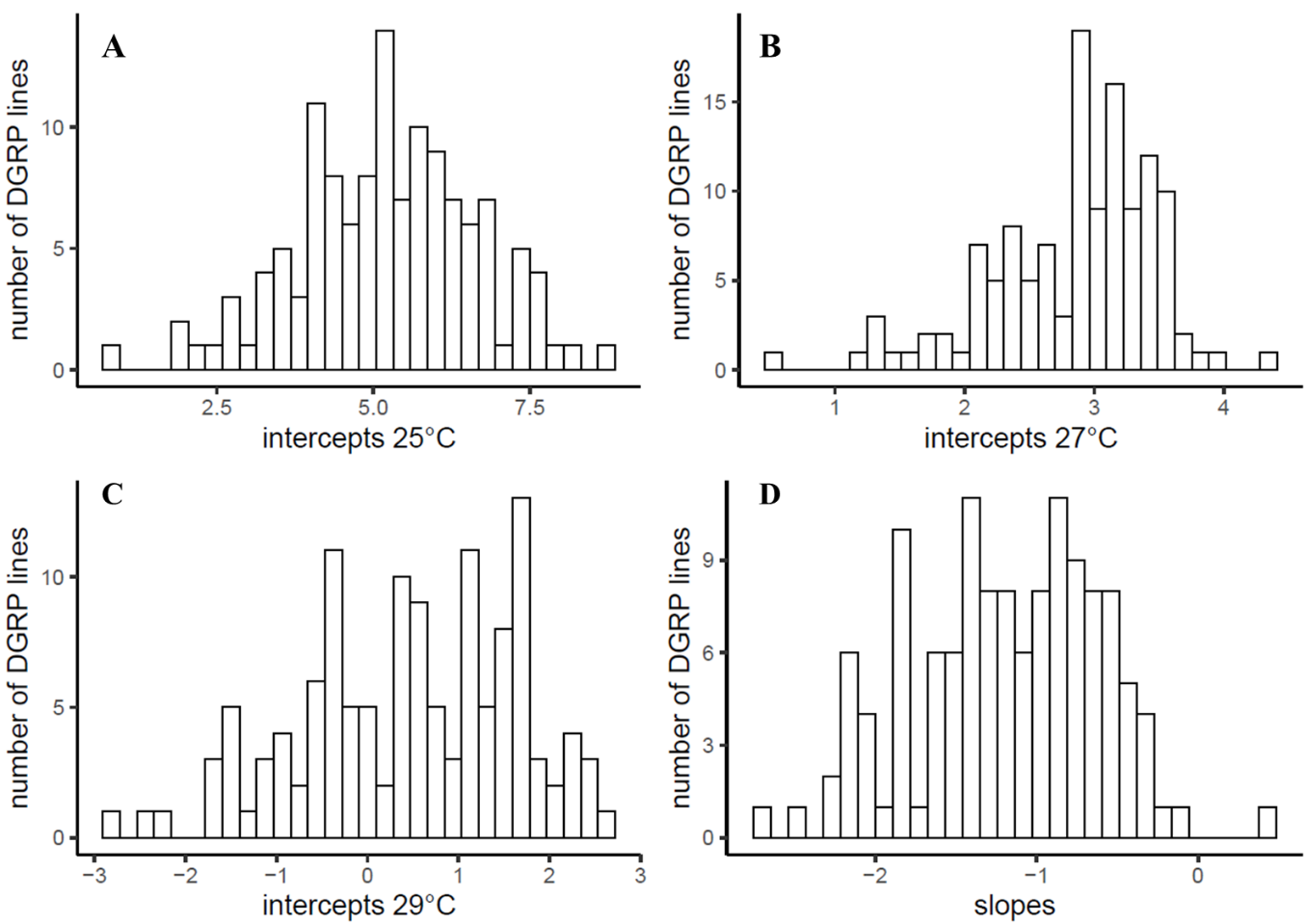
**

**Supplementary Figure 2** Frequency distribution of intercept and slope values for the number of DGRP lines tested for male fertility following developmental heat stress at **A** 25°C **B** 27°C **C** 29°C, and **D** plasticity of response.

**
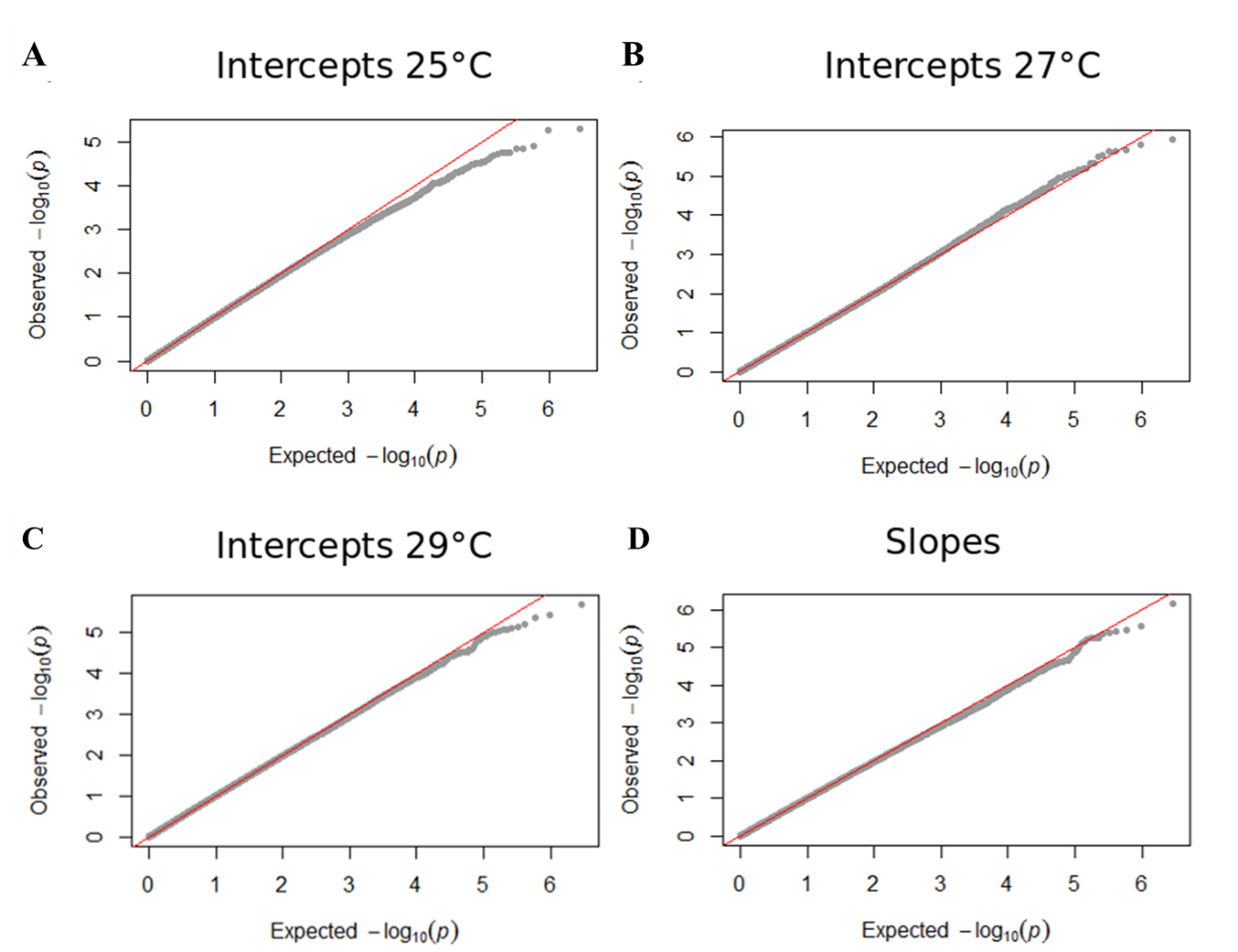
**

**Supplementary Figure 3** Quantile-Quantile plots showing the distribution of –log(10) P values of single nucleotide polymorphisms (SNPs) tested for association with male fertility following developmental heat stress at **A** 25°C **B** 27°C **C** 29°C, and **D** plasticity of response.

**Supplementary Table 1** Excel data file with all datasets analyzed in this paper. This table is provided in a separate supplemental file. Data file with data analyzed for this paper, containing five tabs; the first tab (Legend) explains the contents of the remaining four tabs, each of which contains relevant data for the paper. Datasets and respective descriptions are also available at  <https://doi.org/10.17045/sthlmuni.12248576.v1>

**Supplementary Table 2** Correlation and p values across three replicates (Block A, B, C) of the same subset of DGRP lines (n = 40) for the proportion of male fertility at each developmental heat stress temperature of 25°C, 27°C and 29°C. Block A data are from the large experiment reported here, Blocks B and C data are replicates from a second experiment (ms in preparation).

| Temperature | Variables | Correlation | P value |
| --- | --- | --- | --- |
| 25°C | Block A, Block B | 0.24 | 0.17 |
|  | Block B, Block C | -0.15 | 0.43 |
|  | Block A, Block C | 0.00 | 0.99 |
|  |  |  |  |
| 27°C | Block A, Block B | 0.42 | 0.02 |
|  | Block B, Block C | 0.55 | 0.00 |
|  | Block A, Block C | 0.45 | 0.01 |
|  |  |  |  |
| 29°C | Block A, Block B | 0.73 | 0.00 |
|  | Block B, Block C | 0.88 | 0.00 |
|  | Block A, Block C | 0.78 | 0.00 |

**Supplementary Table 3** Generalized linear mixed-effect model for male reproductive performance (binomial: number of successfully reproducing males in all trials/DGRP line) following development heat stress at 25°C (A), 27°C (B) and 29°C (C), used to extract continuous variables of line-specific slopes and intercepts for GWAS analysis. For each intercept (A,B,C), temperature is centered at that temperature. The correlation column indicates a correlation between random intercepts and random slopes for the DGRP lines.

|  | | | | | | | | | | | | | | |
| --- | --- | --- | --- | --- | --- | --- | --- | --- | --- | --- | --- | --- | --- | --- |
|  | **A. 25°C** | | | | **B. 27°C** | | | | | **C. 29°C** | | | | |
| *Fixed effects* |  |  |  |  |  |  | |  |  |  |  | |  |  |
|  | **Estimate** | **Std. Error** | **Z value** | **P value** | **Estimate** | **Std. Error** | | **Z value** | **P value** | **Estimate** | **Std. Error** | | **Z value** | **P value** |
| Intercept | 5.36 | 0.38 | 14.21 | <0.001 | 2.87 | 0.31 | | 9.18 | <0.001 | 0.38 | 0.32 | | 1.17 | 0.24 |
| Temperature | -1.25 | 0.08 | -15.71 | <0.001 | -1.25 | 0.08 | | -15.71 | <0.001 | -1.25 | 0.08 | | -15.71 | <0.001 |
| *Random effects* |  |  |  |  |  |  | |  |  |  |  | |  |  |
|  | **Variance** | | **Correlation** | | **Variance** | | **Correlation** | | | **Variance** | | **Correlation** | | |
| Line [Intercept] | 3.58 | |  | | 0.80 | |  | | | 1.94 | |  | | |
| Line [Slope] | 0.49 | | -0.89 | | 0.49 | | -0.33 | | | 0.49 | | 0.79 | | |
| Block [Intercept] | 1.09 | |  | | 1.09 | |  | | | 1.09 | |  | | |

**Supplementary Table 4** Variance components used to estimate broad-sense heritability (H^2^) of male fertility following developmental heat stress at 25°C (A), 27°C (B) and 29°C (C) (H^2^ reported in Table 2). Residual variance of the model = 3.29 (Nakagawa and Schielzeth, 2010).

|  | | | | | | | | | | | | | |
| --- | --- | --- | --- | --- | --- | --- | --- | --- | --- | --- | --- | --- | --- |
|  | **A. 25°C** | | | | **B. 27°C** | | | | **C. 29°C** | | | | |
| *Fixed effects* |  | | | |  | | | |  | | | | |
|  | **Estimate** | **Std. Error** | **Z value** | **P value** | **Estimate** | **Std. Error** | **Z value** | **P value** | **Estimate** | | **Std. Error** | **Z value** | **P value** |
| Intercept | 3.91 | 0.30 | 13.16 | <0.001 | 3.30 | 0.25 | 13.25 | <0.001 | 0.21 | | 0.39 | 0.55 | 0.58 |
|  |  |  |  |  |  |  |  |  |  | |  |  |  |
| *Random effects* |  |  |  |  |  |  |  |  |  | |  |  |  |
|  | **Variance** | | | | **Variance** | | | | **Variance** | | | | |
| Block | 0.68 | | | | 0.49 | | | | 1.61 | | | | |
| Line | 0.89 | | | | 1.00 | | | | 2.55 | | | | |
|  |  | | | |  | | | | |  | | | |
|  | | | | | | | | | | | | | |

**Supplementary Table 5** Nominally significant SNPs associated with male sterility after heat stress at three different developmental temperatures (intercept at 25°C, 27°C, 29°C) and the slope of response, the site class and FlyBase gene numbers.

| SNP/Indel | Trait | P value | Site class | FlyBase Gene ID |
| --- | --- | --- | --- | --- |
| 2L_7449491_SNP | Intercept 25°C | 5.15e-06 | Intron | FBgn0025697 |
| 3L_5973068_SNP | Intercept 25°C | 5.34e-06 |  |  |
| 2L_10048933_SNP | Intercept 27°C | 2.18e-06 | Upstream | FBgn0053301 |
| 2L_16175785_SNP | Intercept 27°C | 6.36e-06 | Synonymous coding | FBgn0001991 |
| 2L_2499817_SNP | Intercept 27°C | 3.08e-06 | Donwstream | FBgn0011818 |
| 2L_6112786_SNP | Intercept 27°C | 4.63e-06 | Intron | FBgn0266521 |
| 2R_14285628_SNP | Intercept 27°C | 2.33e-06 | Intron, Upstream | FBgn0063499, FBgn0034335 |
| 2R_16335992_SNP | Intercept 27°C | 2.80e-06 | Intron | FBgn0086604 |
| 2R_2503917_SNP | Intercept 27°C | 9.13e-06 | Synonymous coding | FBgn0086655 |
| 2R_4687576_SNP | Intercept 27°C | 7.64e-06 | Intron | FBgn0024189 |
| 2R_6733675_SNP | Intercept 27°C | 1.58e-06 | Intron | FBgn0050015 |
| 2R_9240476_SNP | Intercept 27°C | 6.95e-06 | Intron, Downstream | FBgn0265104, FBgn0033828 |
| 2R_9661629_SNP | Intercept 27°C | 8.09e-06 | Intron | FBgn0033867 |
| 3L_15222764_SNP | Intercept 27°C | 9.02e-06 |  |  |
| 3L_15224069_DEL | Intercept 27°C | 6.74e-06 |  |  |
| 3L_4745616_SNP | Intercept 27°C | 8.34e-06 | Intron, Downstream | FBgn0035574, FBgn0264474 |
| 3L_6496640_SNP | Intercept 27°C | 9.55e-06 | Intron | FBgn0020251 |
| 3L_7983165_SNP | Intercept 27°C | 2.38e-06 | Intron | FBgn0011817 |
| 3L_8066144_SNP | Intercept 27°C | 4.76e-06 | Intron | FBgn0262579 |
| 3R_10978589_SNP | Intercept 27°C | 1.16e-06 | Intron | FBgn0038295 |
| 3R_14120543_SNP | Intercept 27°C | 8.66e-06 | Intron | FBgn0010877 |
| 3R_22135453_SNP | Intercept 27°C | 6.38e-06 |  |  |
| X_5549867_SNP | Intercept 27°C | 7.76e-06 | Intron | FBgn0259150 |
| 3L_20027412_SNP | Intercept 29°C | 4.40e-06 |  |  |
| 3R_24262915_SNP | Intercept 29°C | 8.49e-06 | Intron | FBgn0039584 |
| 3R_24690741_SNP | Intercept 29°C | 6.38e-06 | Intron | FBgn0264324 |
| 3R_24694269_SNP | Intercept 29°C | 8.71e-06 | Synonymous coding | FBgn0264324 |
| X_12934235_SNP | Intercept 29°C | 7.97e-06 | Intron | FBgn0265597 |
| X_12934291_SNP | Intercept 29°C | 9.38e-06 | Intron | FBgn0265597 |
| X_3335245_SNP | Intercept 29°C | 2.05e-06 | Intron | FBgn0029657, FBgn0266350 |
| 2L_10432357_SNP | Intercept 29°C, Slope | 8.41e-06 | Synonymous coding, Downstream | FBgn0004915, FBgn0032249 |
| 2R_8937890_SNP | Intercept 29°C, Slope | 3.65e-06 | Upstream | FBgn0033789 |
| 3L_7931396_SNP | Intercept 29°C, Slope | 7.42e-06 |  |  |
| 2L_10431904_SNP | Slope | 5.55e-06 | Intron, Downstream | FBgn0004915, FBgn0032249 |
| 2L_12325475_SNP | Slope | 7.13e-06 |  |  |
| 2R_8937917_SNP | Slope | 2.65e-06 | Upstream | FBgn0033789 |
| 3L_7931409_SNP | Slope | 3.81e-06 |  |  |
| 3L_7931410_SNP | Slope | 3.88e-06 |  |  |
| 3L_7931436_SNP | Slope | 4.33e-06 |  |  |
| 3R_17172017_SNP | Slope | 9.66e-06 | Intron | FBgn0038881 |
| 3R_17172690_SNP | Slope | 6.00e-06 | Intron | FBgn0038881 |
| 3R_20979624_SNP | Slope | 5.37e-06 |  |  |
| 3R_24702747_SNP | Slope | 5.77e-06 | Intron, Synonymous coding | FBgn0053203, FBgn0039611 |
